# Ventilation inhibits sympathetic action potential recruitment even during severe chemoreflex stress

**DOI:** 10.1152/jn.00381.2017

**Published:** 2017-08-23

**Authors:** Mark B. Badrov, Otto F. Barak, Tanja Mijacika, Leena N. Shoemaker, Lindsay J. Borrell, Mihajlo Lojpur, Ivan Drvis, Zeljko Dujic, J. Kevin Shoemaker

**Affiliations:** ^1^School of Kinesiology, Western University, London, Ontario, Canada;; ^2^Department of Physiology, University of Split School of Medicine, Split, Croatia;; ^3^Faculty of Medicine, University of Novi Sad, Novi Sad, Serbia;; ^4^Faculty of Kinesiology, University of Zagreb, Zagreb, Croatia; and; ^5^Department of Physiology and Pharmacology, Western University, London, Ontario, Canada

**Keywords:** action potential, chemoreflex, muscle sympathetic nerve activity

## Abstract

This study investigated the influence of ventilation on sympathetic action potential (AP) discharge patterns during varying levels of high chemoreflex stress. In seven trained breath-hold divers (age 33 ± 12 yr), we measured muscle sympathetic nerve activity (MSNA) at baseline, during preparatory rebreathing (RBR), and during *1*) functional residual capacity apnea (FRC_Apnea_) and *2*) continued RBR. Data from RBR were analyzed at matched (i.e., to FRC_Apnea_) hemoglobin saturation (HbSat) levels (RBR_Matched_) or more severe levels (RBR_End_). A third protocol compared alternating periods (30 s) of FRC and RBR (FRC-RBR_ALT_). Subjects continued each protocol until 85% volitional tolerance. AP patterns in MSNA (i.e., providing the true neural content of each sympathetic burst) were studied using wavelet-based methodology. First, for similar levels of chemoreflex stress (both HbSat: 71 ± 6%; *P* = NS), RBR_Matched_ was associated with reduced AP frequency and APs per burst compared with FRC_Apnea_ (both *P* < 0.001). When APs were binned according to peak-to-peak amplitude (i.e., into clusters), total AP clusters increased during FRC_Apnea_ (+10 ± 2; *P* < 0.001) but not during RBR_Matched_ (+1 ± 2; *P* = NS). Second, despite more severe chemoreflex stress during RBR_End_ (HbSat: 56 ± 13 vs. 71 ± 6%; *P* < 0.001), RBR_End_ was associated with a restrained increase in the APs per burst (FRC_Apnea_: +18 ± 7; RBR_End_: +11 ± 5) and total AP clusters (FRC_Apnea_: +10 ± 2; RBR_End_: +6 ± 4) (both *P* < 0.01). During FRC-RBR_ALT_, all periods of FRC elicited sympathetic AP recruitment (all *P* < 0.001), whereas all periods of RBR were associated with complete withdrawal of AP recruitment (all *P* = NS). Presently, we demonstrate that ventilation per se restrains and/or inhibits sympathetic axonal recruitment during high, and even extreme, chemoreflex stress.

**NEW & NOTEWORTHY** The current study demonstrates that the sympathetic neural recruitment patterns observed during chemoreflex activation induced by rebreathing or apnea are restrained and/or inhibited by the act of ventilation per se, despite similar, or even greater, levels of severe chemoreflex stress. Therefore, ventilation modulates not only the timing of sympathetic bursts but also the within-burst axonal recruitment normally observed during progressive chemoreflex stress.

## Abstract (precis)

The current study demonstrates that the sympathetic neural recruitment patterns observed during chemoreflex activation induced by rebreathing or apnea are restrained and/or inhibited by the act of ventilation per se, despite similar, or even greater, levels of severe chemoreflex stress. Therefore, ventilation modulates not only the timing of sympathetic bursts but also the within-burst axonal recruitment normally observed during progressive chemoreflex stress.

the chemoreflex represents a highly coordinated and integrative pattern of response to systemic hypoxia and/or hypercapnia that ultimately regulates and defends perfusion to critical organs and tissues within the body. As such, the homeostatic challenges presented by central and/or peripheral chemoreceptor stimulation are corrected through reflexive increases in ventilation, as well as autonomic circulatory arousal (Guyenet 2014). Indeed, the compensatory response to chemoreflex activation involves large elevations in efferent sympathetic nerve outflow directed to skeletal muscle (i.e., muscle sympathetic nerve activity, MSNA) and other vascular beds (Leuenberger et al. 2005; Malpas and Ninomiya 1992a, 1992b; Morgan et al. 1995; Saito et al. 1988). Furthermore, the sympathetic response to voluntary apnea and/or pathological apnea (i.e., obstructive sleep apnea) is large (Hardy et al. 1994; Leuenberger et al. 1995; Morgan et al. 1993; Steinback et al. 2010b) and, in turn, has been linked to poor clinical outcomes (Kara et al. 2003).

The mechanisms mediating the robust sympathetic response to apnea remain uncertain and appear to be diverse. Indeed, one fundamental issue is the identification of the specific roles played by the ventilatory response (or the lack thereof in the case of volitional apnea) vs. the chemoreflex activation itself. For example, voluntary apnea potentiated the sympathetic response to isocapnic hypoxia, indicating the sympathoinhibitory nature of ventilation (Somers et al. 1989a, 1989b). Furthermore, the chemoreflex stimulus to typical 20- to 30-s apneas in untrained breath-hold performers is rather unremarkable (Heusser et al. 2009; Leuenberger et al. 2005; Seitz et al. 2013), yet the MSNA response is large; therefore, factors other than chemoreflex stress must be involved in eliciting sympathetic activation. The possible factors affecting the sympathoinhibitory effect of ventilation during chemoreflex stimulation may include central pathways that coordinate respiratory drive with sympathetic inhibition. Evidence for such pathways has been difficult to find in humans (St Croix et al. 1999), whereby MSNA is inhibited during inspiration (or at high lung volumes) and increases during expiration (or at low lung volumes) (Eckberg et al. 1985; Seals et al. 1993); however, current evidence in the intact human argues against a significant role played by central respiratory drive on the ventilatory modulation of MSNA (Seals et al. 1993; St Croix et al. 1999). In contrast, such pathways can be induced in anesthetized rodents (Darnall and Guyenet 1990; Haselton and Guyenet 1989; Numao et al. 1987), leading Guyenet and colleagues (Guyenet 2014; Guyenet et al. 2010) to speculate that species differences exist. Nonetheless, current evidence suggests that the sympathetic response to apnea has more to do with the lack of breathing rather than the chemoreflex stimulus per se.

Much remains uncertain regarding the interactions between chemoreflex stress and ventilation on sympathetic neurocirculatory control. For example, the degree of chemoreflex stress may be an important caveat in understanding the role of respiratory movements in sympathetic control. The central motor hypothesis has been studied in humans mainly under normoxic and normocapnic conditions (St Croix et al. 1999) or low levels of hypercapnia (Seals et al. 1993), raising the possibility that potentiation of central pathways associated with the drive to breathe and sympathetic inhibition with breathing may be augmented with concurrent chemoreflex activation. Observations arguing against this possibility are provided from our earlier study (Steinback et al. 2010a), in which the very large sympathetic responses occurring during prolonged apnea at functional residual capacity (FRC), performed by elite breath-hold divers, were acutely inhibited by a two-breath rebreathe protocol performed immediately following the apnea, despite a sustained chemoreflex stimulus. However, the acute vs. chronic effects were not studied, nor was it determined whether this has to do with reduction of the emotional component of resisting the breathing movements, the absence of lung-stretch during apnea, or the actual degree of chemoreflex stimulus (Steinback et al. 2010a).

In addition, the mode of studying sympathetic nerve activity likely has an impact on determinations of chemoreflex vs. ventilatory control over sympathetic nerve recruitment. Indeed, early work from single-unit action potential (AP) recordings demonstrated that the sympathetic adjustment to apnea involves not only an increase in neural firing probability but also a shift toward greater within-burst firing of single axons (Macefield and Wallin 1999). Therefore, the content and variability of sympathetic APs within and between integrated bursts more accurately represents the neural signal and, ultimately, reflects the neural discharge and recruitment patterns used by the central nervous system to fine-tune sympathetic outflow. The need to study AP behavior becomes especially relevant when one considers that burst frequency and burst size are regulated differently. Specifically, burst frequency reflects the rate and probability of sympathetic discharge, whereas burst size reflects the number and/or size of recruited APs (Ninomiya et al. 1993; Salmanpour et al. 2011). Chemoreflex stress appears to exert preferential influence on burst size (Lim et al. 2015; Malpas et al. 1996). Using a multiunit AP approach, we have found that the central recruitment strategies used to alter sympathetic drive in response to apneic stress involve both an increased firing of previously recruited axons and recruitment of subpopulations of larger, latent axons (Badrov et al. 2015, 2016a; Breskovic et al. 2011; Steinback et al. 2010b). However, whether neural recruitment is restrained and/or inhibited in the presence of ventilation remains unknown.

Therefore, the objective of the current investigation was to determine the influence of ventilation on sympathetic AP recruitment during varying levels of high chemoreflex stress. To do so, we studied sympathetic AP discharge during apnea at FRC (i.e., absence of ventilation) and during asphyxic rebreathing (i.e., presence of ventilation) in trained breath-hold divers. Trained breath-hold divers were studied because of their unique ability to perform apneas for prolonged periods of time and their capacity to endure high levels of chemoreflex stress (Heusser et al. 2009). We tested the hypothesis that the sympathoinhibitory effect of ventilation on chemoreflex-induced sympathetic activation is limited to mild chemoreflex stress. If so, then it can be expected that for similar levels of chemoreflex stimuli, the presence of ventilation during rebreathing would restrain sympathetic AP recruitment compared with apnea. However, during more severe rebreathe-induced chemoreflex levels, the inhibitory influence of ventilation would be overridden such that AP recruitment would be similar to that observed during apnea.

## METHODS

### 

#### Participants.

Seven healthy individuals (1 female; 33 ± 12 yr, 181 ± 10 cm, 74 ± 12 kg, body mass index = 22 ± 2 kg/m^2^) participated in the current investigation after providing informed written consent. Participants were nonsmokers without any history of cardiovascular or respiratory disease. All studies were conducted at the University of Split, adhering to the standards set by the latest revision of the Declaration of Helsinki, and all experimental procedures were approved by the Research Ethics Board at the University of Split School of Medicine in Croatia.

#### Experimental protocol.

All experiments were conducted following a 3-h fast and a 12-h abstinence from caffeine, alcohol, and vigorous exercise. Participants voided their bladder immediately prior to the study. All testing was completed in the supine position. Participants were fitted with a mouthpiece (series 9060; Hans Rudolph, Kansas City, MO) connected to a three-way valve, allowing them to breathe either room air, or through a Y-connector (VacuMed, Ventura, CA) leading to two 3-liter breathing bags. An infrared probe connected to a pulse oximeter (Poet II; Criticare Systems, Waukesha, WI) was applied to the index finger to monitor arterial oxygen saturation (i.e., hemoglobin saturation, HbSat). Respiratory gases were analyzed using an infrared carbon dioxide sensor and optical oxygen detector fed from a damped micro-vacuum sampling pump (ML206 gas analyzer; ADInstruments, Colorado Springs, CO) and calibrated using ambient air pressure values, which were converted to online measurements of oxygen (Po_2_) and carbon dioxide (Pco_2_) partial pressures. End-tidal values for Po_2_ ($PET_{O_{2}}$) and Pco_2_ ($PET_{CO_{2}}$) were acquired using peak parameters software (LabChart7; ADInstruments).

Participants completed three protocols in random order, each separated by 5 min of recovery. Before the onset of each protocol, participants expired into the breathing bags to fill them with air for an ensuing rebreathing period. The first protocol (i.e., apnea protocol) assessed the impact of high levels of chemoreflex stress on sympathetic axonal recruitment in the absence of ventilation. Specifically, following 3 min of baseline data collection, the three-way valve was turned at end-expiration to begin an initial period of rebreathing, in an attempt to maximize the severity of chemoreflex stimulation incurred during the ensuing apnea period (Badrov et al. 2015; Usselman et al. 2013). This initial rebreathe period continued until $PET_{O_{2}}$ reached 70 Torr, at which point participants performed an end-expiratory apnea at FRC until they achieved ~85% of their self-perceived “maximal” tolerance for the maneuver. Upon cessation, participants breathed twice into and out of the breathing bags for the measurement of end-apnea $PET_{O_{2}}$ and $PET_{CO_{2}}$. Average apnea duration in the current study was 89 ± 18 s. The second protocol (i.e., rebreathing protocol) assessed the impact of chemoreflex stress on sympathetic axonal recruitment in the presence of ventilation. Specifically, following 3 min of baseline data collection, the three-way valve was turned to initiate rebreathing and participants were instructed to rebreathe until, once again, they achieved ~85% of their self-perceived maximal tolerance for the maneuver. Finally, a third protocol (FRC-RBR_ALT_ protocol; *n* = 6) was completed in which baseline data were collected for 2 min, followed by alternating 30-s periods of FRC apnea and rebreathing until ~85% maximal tolerance.

#### Experimental measures.

Multiunit recordings of MSNA were obtained from the right peroneal nerve by microneurography (Hagbarth and Vallbo 1968) (model 662C-3, Nerve Traffic Analyzer; Dept. of Bioengineering, University of Iowa, Iowa City, IA), using standard methodology described in detail elsewhere (Badrov et al. 2015). Heart rate (HR) was determined from a standard three-lead electrocardiogram. Continuous beat-to-beat blood pressure was obtained throughout all protocols using finger photoplethysmography (Finometer; Finapres Medical Systems, Amsterdam, The Netherlands), the values of which were calibrated to manual sphygmomanometry values taken at baseline. Stroke volume (SV) and cardiac output (CO) were determined using the Finometer Modelflow algorithm (Wesseling et al. 1993), and total peripheral resistance (TPR) was calculated as the quotient of mean arterial pressure (MAP) and CO. All data were collected using LabChart7 and the PowerLab data acquisition system (ADInstruments).

#### Data analysis.

As a first aim, the study addressed the effect of the “absence or presence” of ventilation on sympathetic neural recruitment during similar (i.e., matched) levels of chemoreflex stress. To address this aim, data were analyzed from the apnea protocol for the 3-min baseline period, the rebreathe period (i.e., until 70 Torr $PET_{O_{2}}$), and the second half of the FRC apnea maneuver (i.e., FRC_Apnea_). For the rebreathe protocol, data were analyzed for the 3-min baseline period, for the initial rebreathe period (i.e., until 70 Torr $PET_{O_{2}}$), and during the period of continued rebreathe that corresponded to the same HbSat levels incurred during the second half of FRC_Apnea_ (i.e., RBR_Matched_). Therefore, this represents the FRC_Apnea_ vs. RBR_Matched_ comparison at the same level of chemoreflex stress, as inferred from the HbSat values (i.e., because $PET_{CO_{2}}$ could not be measured in the FRC_Apnea_ component).

The second aim determined the influence of ventilation on sympathetic neural recruitment at more severe levels of chemoreflex stress than that induced by FRC_Apnea_. Therefore, we compared data from the second half of the FRC_Apnea_ protocol, as above, with data observed at the end of the continued rebreathe protocol (i.e., RBR_End_). The duration of data studied in the RBR_End_ period was matched to that of the last half of the FRC_Apnea_, used in the first aim. Therefore, this represents the FRC_Apnea_ vs. RBR_End_ comparison.

A third aim addressed the issue that the apnea or continued rebreathe protocols always occurred at the end of each protocol, and therefore, we compared alternating periods of FRC apnea with rebreathe during progressively increasing chemoreflex stress. For the FRC-RBR_ALT_ protocol, because the number of alternating FRC apnea and rebreathe periods differed between participants, the 2-min baseline and the final three FRC apnea and rebreathe periods for each participant were used for analysis.

MSNA data were analyzed in two manners. First, the integrated neurogram was quantified as per traditional methodology. Second, we studied the patterns of change in APs from the filtered raw MSNA signal as per our approach detailed previously (Salmanpour et al. 2010). Integrated sympathetic bursts were identified as exhibiting a pulse-synchronous burst pattern, having a signal-to-noise ratio of at least 2:1 with respect to the previous period of neural silence, presenting with characteristic rising and falling slopes, and having APs visible in the corresponding raw and filtered neurograms. Integrated sympathetic activity was quantified using burst frequency (the number of bursts per minute), burst incidence (the number of bursts per 100 heartbeats; hb), burst amplitude (normalized to the largest burst recorded at baseline, which was given a value of 100), and total MSNA (the product of normalized burst amplitude and burst frequency).

For the analysis of sympathetic AP patterns, wavelet-based methodology was used to detect and extract APs from the filtered raw MSNA signal (Salmanpour et al. 2010). Specifically, as described in detail previously (Badrov et al. 2015; Salmanpour et al. 2011; Steinback et al. 2010b), a continuous wavelet transform was used, in which a “mother wavelet” (adapted from actual physiological recordings of postganglionic sympathetic APs) was applied to the filtered raw MSNA neurogram to identify and extract APs at their point of occurrence. Next, extracted APs were ordered on the basis of their peak-to-peak amplitude into “clusters” (i.e., bins of similarly sized APs) (Scott 1979). Within participants, cluster characteristics were normalized within (i.e., baseline to rebreathe to maneuver) and between (i.e., FRC_Apnea_ vs. RBR_Matched_ vs. RBR_End_) protocols to ensure that bin width, maximum bin center, and the total number of bins would be identical across conditions. This procedure ensured that corresponding clusters within and between protocols contain APs with the same peak-to-peak amplitude. Therefore, within protocols (i.e., baseline to rebreathe to maneuver), an increase in the number of total clusters represents recruitment of latent subpopulations of larger sized axons not present at baseline, whereas between protocols (i.e., FRC_Apnea_ vs. RBR_Matched_ vs. RBR_End_), a greater number of total clusters represents further recruitment of these latent subpopulations compared with other protocols. Sympathetic AP patterns were quantified using AP frequency (the number of APs per minute), whereas sympathetic AP recruitment was expressed using the mean AP content per integrated sympathetic burst (APs/burst), the number of active AP clusters per integrated sympathetic burst (clusters/burst), and the number of total AP clusters (total clusters). Based on previous validation analysis of our technique (Salmanpour et al. 2010), the average signal-to-noise ratio in the current study (i.e., 4.8 ± 0.6) is expected to produce a correct AP detection rate of >90% and a false positive rate of <3%.

#### Statistical analysis.

All statistical analyses were performed using SigmaPlot 12.0 (Systat Software, San Jose, CA). All data were normally distributed as confirmed by Shapiro-Wilk tests. Two-way repeated-measures ANOVA was used to assess the effect of protocol (i.e., FRC_Apnea_ vs. RBR_Matched_; FRC_Apnea_ vs. RBR_End_) vs. time (i.e., baseline vs. rebreathe vs. maneuver). For the FRC-RBR_ALT_ protocol, one-way repeated-measures ANOVA was used to assess the effect of time (i.e., baseline vs. FRC vs. RBR). Bonferroni-corrected post hoc procedures were used to assess specific differences between means, when appropriate. Statistical significance was set at *P* < 0.05, and all data are means ± SD.

## RESULTS

**Fig. 1.**
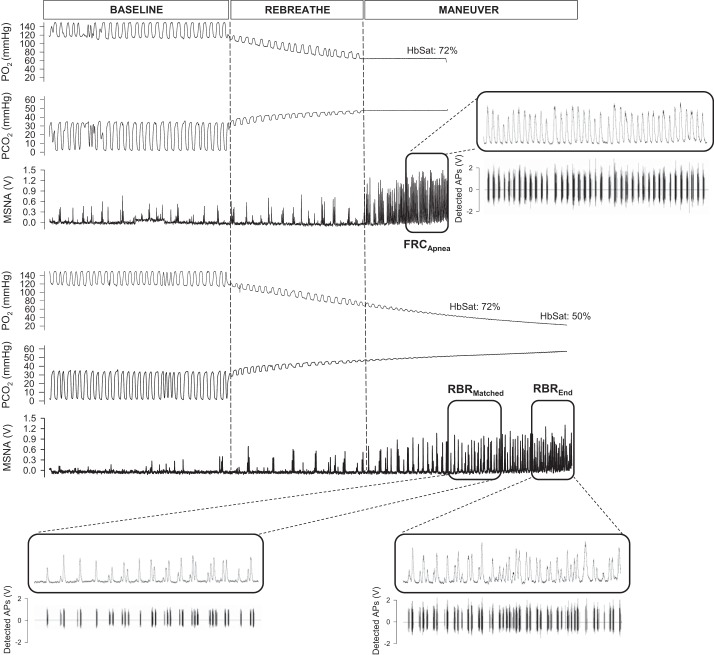
Representative recordings of the integrated muscle sympathetic nerve activity (MSNA) neurogram and detected action potentials (APs) (and associated chemoreflex stimuli) from one individual during the functional residual capacity (FRC) apnea and rebreathing (RBR) protocols. Po_2_, partial pressure of oxygen; Pco_2_, partial pressure of carbon dioxide; HbSat, hemoglobin saturation. Highlighted (i.e., boxed) areas represent portions of the maneuver used for analysis.

Figure 1 displays representative recordings of the integrated MSNA neurogram and detected APs (and associated chemoreflex stimuli) from one individual during the FRC apnea and rebreathing protocols.

### 

#### FRC_Apnea_ vs. RBR_Matched_.

Both FRC_Apnea_ and RBR_Matched_ elicited large reductions in HbSat (Table 1; *P* < 0.001) at end-maneuver. By design, HbSat levels were similar (i.e., matched) between maneuvers (71 ± 6 vs. 71 ± 6%; *P* = NS). Compared with baseline, $PET_{CO_{2}}$ (FRC_Apnea_: +17 ± 2 Torr; RBR_Matched_: +19 ± 2 Torr; both *P* < 0.001) increased, whereas $PET_{O_{2}}$ (FRC_Apnea_: −68 ± 7 Torr; RBR_Matched_: −84 ± 7 Torr; both *P* < 0.001) decreased, during FRC_Apnea_ and RBR_Matched_.

**Table 1.** Hemodynamic responses to FRC_Apnea_ and RBR_Matched_ protocols

	Baseline	Rebreathe	Maneuver
MAP, mmHg			
FRC_Apnea_	87 ± 3	89 ± 3	105 ± 9*†
RBR_Matched_	87 ± 3	88 ± 2	97 ± 6*
HR, beats/min			
FRC_Apnea_	70 ± 14	68 ± 13	60 ± 9*†
RBR_Matched_	68 ± 12	66 ± 13	75 ± 19*
SV, ml			
FRC_Apnea_	97 ± 20	90 ± 18	84 ± 18
RBR_Matched_	96 ± 19	91 ± 19	86 ± 24
CO, l/min			
FRC_Apnea_	6.6 ± 1.3	6.0 ± 1.0	5.0 ± 0.7*†
RBR_Matched_	6.4 ± 0.9	5.9 ± 0.8	6.1 ± 0.9
TPR, mmHg·l^−1^·min^−1^			
FRC_Apnea_	13.5 ± 2.3	15.1 ± 2.1	21.6 ± 3.3*†
RBR_Matched_	13.7 ± 1.7	15.2 ± 1.9	16.1 ± 2.8*
HbSat, %			
FRC_Apnea_	99.5 ± 0.3	97.2 ± 1.5	71.3 ± 6.3
RBR_Matched_	99.5 ± 0.4	97.5 ± 1.8	71.3 ± 6.3
$PET_{CO_{2}}$, Torr			
FRC_Apnea_	27 ± 6	41 ± 5*	44 ± 4*†
RBR_Matched_	28 ± 6	40 ± 6*	47 ± 5*
$PET_{O_{2}}$, Torr			
FRC_Apnea_	118 ± 5	66 ± 5*	50 ± 5*†
RBR_Matched_	117 ± 7	68 ± 6*	33 ± 3*

Values are means ± SD. MAP, mean arterial pressure; HR, heart rate; SV, stroke volume; CO, cardiac output; TPR, total peripheral resistance; HbSat, hemoglobin saturation; $PET_{CO_{2}}$, end-tidal partial pressure of carbon dioxide; $PET_{O_{2}}$, end-tidal partial pressure of oxygen. Effects of time were found for SV and HbSat whereby the maneuver periods were reduced vs. baseline (both *P* ≤ 0.001).

* *P* < 0.05, significantly different from baseline.

† *P* < 0.05, significantly different from RBR_Matched_.

Integrated MSNA indexes during the baseline, rebreathe, and maneuver periods of the FRC_Apnea_ and RBR_Matched_ protocols are outlined in Table 2. Specifically, compared with baseline, burst frequency (FRC_Apnea_: +29 ± 7 bursts/min; RBR_Matched_: +12 ± 9 bursts/min; both *P* < 0.01) and burst incidence (FRC_Apnea_: +52 ± 16 bursts/100 hb; RBR_Matched_: +14 ± 12 bursts/100 hb; both *P* < 0.05) increased during both maneuvers; however, values of burst frequency and burst incidence were greater during FRC_Apnea_ than during RBR_Matched_ (both *P* < 0.001). Similarly, burst amplitude (FRC_Apnea_: +165 ± 85%; RBR_Matched_: +69 ± 55%; both *P* < 0.01) increased above baseline levels during both maneuvers, although levels were greater during FRC_Apnea_ (*P* < 0.001). As such, compared with baseline, total MSNA was elevated during both maneuver periods (FRC_Apnea_: +695 ± 397%; RBR_Matched_: +336 ± 481%; both *P* < 0.05), yet, once again, levels of total MSNA were greater during FRC_Apnea_ (*P* < 0.001).

**Table 2.** Integrated MSNA responses to FRC_Apnea_ and RBR_Matched_ protocols

	Baseline	Rebreathe	Maneuver
Burst frequency, bursts/min			
FRC_Apnea_	17 ± 5	17 ± 6	46 ± 8*†
RBR_Matched_	16 ± 7	17 ± 6	28 ± 7*
Burst incidence, bursts/100 hb			
FRC_Apnea_	25 ± 10	26 ± 11	77 ± 15*†
RBR_Matched_	24 ± 13	26 ± 11	38 ± 11*
Burst amplitude, AU			
FRC_Apnea_	50 ± 10	53 ± 9	127 ± 32*†
RBR_Matched_	51 ± 4	63 ± 15	87 ± 33*
Total MSNA, AU/min			
FRC_Apnea_	832 ± 298	877 ± 251	5,774 ± 1,572*†
RBR_Matched_	780 ± 386	1,019 ± 307	2,496 ± 1,434*

Values are means ± SD. MSNA, muscle sympathetic nerve activity; hb, heart beats; AU, arbitrary units.

* *P* < 0.05, significantly different from baseline.

† *P* < 0.001, significantly different from RBR_Matched_.

**Fig. 2.**
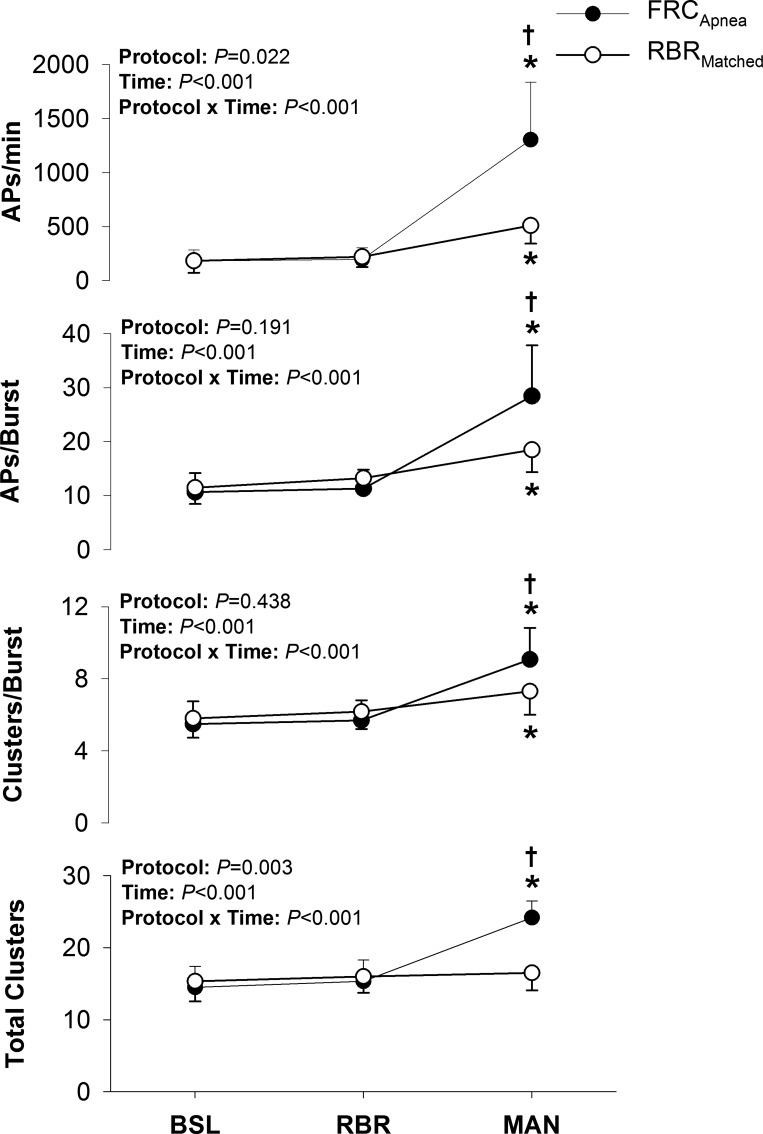
Sympathetic action potential (AP) recruitment during baseline (BSL), initial rebreathe (RBR), and maneuver (MAN) periods of the FRC_Apnea_ and RBR_Matched_ protocols. *P < 0.05, significantly different from BSL. †*P* < 0.01, significantly different from RBR_Matched_.

Figure 2 presents the sympathetic AP discharge indexes during the baseline, rebreathe, and maneuver periods of the FRC_Apnea_ and RBR_Matched_ protocols. Specifically, compared with baseline, AP frequency (FRC_Apnea_: +1,122 ± 534 APs/min; RBR_Matched_: +328 ± 172 APs/min; both *P* < 0.05) increased during both maneuvers; however, AP frequency was greater during FRC_Apnea_ compared with RBR_Matched_ (1,304 ± 534 vs. 511 ± 167 APs/min; *P* < 0.001). This increase in AP frequency was due to the elevated levels of burst frequency but also to an increase in the AP content per integrated sympathetic burst (FRC_Apnea_: +18 ± 7 APs/burst; RBR_Matched_: +7 ± 3 APs/burst; both *P* < 0.01), during both maneuvers, compared with baseline. The AP content per integrated sympathetic burst, however, was greater during FRC_Apnea_ than during RBR_Matched_ (28 ± 9 vs. 18 ± 4 APs/burst; *P* < 0.001). Furthermore, when APs were binned according to peak-to-peak amplitude (i.e., into clusters), the number of total AP clusters increased during FRC_Apnea_ (+10 ± 2 total clusters; *P* < 0.001) but not during RBR_Matched_ (+1 ± 2 total clusters; *P* = NS). As such, the number of total AP clusters was greater during FRC_Apnea_ (*P* < 0.001). Finally, the number of active clusters per burst (FRC_Apnea_: +4 ± 2 clusters/burst; RBR_Matched_: +2 ± 1 clusters/burst; both *P* < 0.01) was elevated above baseline levels during both maneuvers, although levels were greater during FRC_Apnea_ than during RBR_Matched_ (*P* < 0.01).

The hemodynamic responses to both maneuvers were consistent with the sympathetic response patterns as shown in Table 1. Specifically, compared with baseline, MAP (FRC_Apnea_: +18 ± 9 mmHg; RBR_Matched_: +10 ± 7 mmHg; both *P* < 0.01) increased during both maneuvers, yet the pressor response was greater during FRC_Apnea_ (*P* < 0.001). The greater pressor response was associated with a greater TPR during FRC_Apnea_ (*P* < 0.001). HR decreased during FRC_Apnea_ and increased during RBR_Matched_ (both *P* < 0.05), whereas reductions in SV were similar between maneuvers (*P* = NS). Subsequently, compared with baseline, CO decreased during FRC_Apnea_ (*P* < 0.001) but remained unchanged during RBR_Matched_ (*P* = NS).

#### FRC_Apnea_ vs. RBR_End_.

By design, when rebreathing was allowed to continue and analyzed at the end (i.e., RBR_End_), the RBR_End_ maneuver elicited a larger reduction in HbSat vs. FRC_Apnea_ (56 ± 13 vs. 71 ± 6%; *P* < 0.001; Table 3). Similarly, larger increases in $PET_{CO_{2}}$ (FRC_Apnea_: +17 ± 2 Torr; RBR_End_: +21 ± 3 Torr; both *P* < 0.001) and larger decreases in $PET_{O_{2}}$ (FRC_Apnea_: −68 ± 7 Torr; RBR_End_: −94 ± 4 Torr; both *P* < 0.001) were observed during RBR_End_ compared with FRC_Apnea_ (Table 3; both *P* < 0.001).

**Table 3.** Hemodynamic responses to FRC_Apnea_ and RBR_End_ protocols

	Baseline	Rebreathe	Maneuver
MAP, mmHg			
FRC_Apnea_	87 ± 3	89 ± 3	105 ± 9
RBR_End_	87 ± 3	88 ± 2	106 ± 11
HR, beats/min			
FRC_Apnea_	70 ± 14	68 ± 13	60 ± 9†
RBR_End_	68 ± 12	66 ± 13	84 ± 23*
SV, ml			
FRC_Apnea_	97 ± 20	90 ± 18	84 ± 18
RBR_End_	96 ± 19	91 ± 19	87 ± 23
CO, l/min			
FRC_Apnea_	6.6 ± 1.3	6.0 ± 1.0	5.0 ± 0.7*†
RBR_End_	6.4 ± 0.9	5.9 ± 0.8	7.0 ± 1.1
TPR, mmHg·l^−1^·min^−1^			
FRC_Apnea_	13.5 ± 2.3	15.1 ± 2.1	21.6 ± 3.3*†
RBR_End_	13.7 ± 1.7	15.2 ± 1.9	15.5 ± 2.1
HbSat, %			
FRC_Apnea_	99.5 ± 0.3	97.2 ± 1.5	71.3 ± 6.3*†
RBR_End_	99.5 ± 0.4	97.5 ± 1.8	51.9 ± 13.1*
$PET_{CO_{2}}$, Torr			
FRC_Apnea_	27 ± 6	41 ± 5*	44 ± 4*†
RBR_End_	28 ± 6	40 ± 6*	49 ± 6*
$PET_{O_{2}}$, Torr			
FRC_Apnea_	118 ± 5	66 ± 5*	50 ± 5*†
RBR_End_	117 ± 7	68 ± 6*	23 ± 5*

Values are means ± SD. MAP, mean arterial pressure; HR, heart rate; SV, stroke volume; CO, cardiac output; TPR, total peripheral resistance; HbSat, hemoglobin saturation; $PET_{CO_{2}}$, end-tidal partial pressure of carbon dioxide; $PET_{O_{2}}$, end-tidal partial pressure of oxygen. An effect of time was found for SV whereby the maneuver period was reduced vs. baseline (*P* < 0.001). An effect of time was found for MAP whereby the rebreathe and maneuver period were greater than baseline (both P < 0.001).

* *P* < 0.01, significantly different from baseline.

† *P* < 0.01, significantly different from RBR_End_.

Table 4 outlines the integrated MSNA indexes during the baseline, rebreathe, and maneuver periods of the FRC_Apnea_ and RBR_End_ protocols. Compared with baseline, burst frequency (FRC_Apnea_: +29 ± 7 bursts/min; RBR_End_: +32 ± 17 bursts/min; both *P* < 0.001) increased to similar levels during both maneuvers (*P* = NS), whereas burst incidence (FRC_Apnea_: +52 ± 16 bursts/100 hb; RBR_Matched_: +33 ± 17 bursts/100 hb; both *P* < 0.001) increased to a greater extent during FRC_Apnea_ than during RBR_End_ (*P* = 0.001). Burst amplitude (FRC_Apnea_: +165 ± 85%; RBR_End_: +103 ± 46%; both *P* < 0.01) increased above baseline levels during both maneuvers, although levels were greater during FRC_Apnea_ than during RBR_End_ (*P* < 0.05). Finally, compared with baseline, total MSNA was elevated during both maneuver periods (FRC_Apnea_: +695 ± 397%; RBR_End_: +785 ± 903%; both *P* < 0.01); however, levels of total MSNA were not different during FRC_Apnea_ and RBR_End_ (*P* = NS).

**Table 4.** Integrated MSNA responses to FRC_Apnea_ and RBR_End_ protocols

	Baseline	Rebreathe	Maneuver
Burst frequency, bursts/min			
FRC_Apnea_	17 ± 5	17 ± 6	46 ± 8
RBR_End_	16 ± 7	17 ± 6	47 ± 15
Burst incidence, bursts/100 hb			
FRC_Apnea_	25 ± 10	26 ± 11	77 ± 15*†
RBR_End_	24 ± 13	26 ± 11	57 ± 15*
Burst amplitude, AU			
FRC_Apnea_	50 ± 10	53 ± 9	127 ± 32*†
RBR_End_	51 ± 4	63 ± 15	104 ± 29*
Total MSNA, AU/min			
FRC_Apnea_	832 ± 298	877 ± 251	5,774 ± 1,572
RBR_End_	780 ± 386	1,019 ± 307	5,170 ± 2,745

Values are means ± SD. MSNA, muscle sympathetic nerve activity; hb, heart beats; AU, arbitrary units. Effects of time were found for burst frequency and total MSNA, whereby the rebreathe and maneuver periods were greater than baseline (all *P* < 0.001).

* *P* < 0.05, significantly different from baseline.

† *P* < 0.001, significantly different from RBR_End_.

**Fig. 3.**
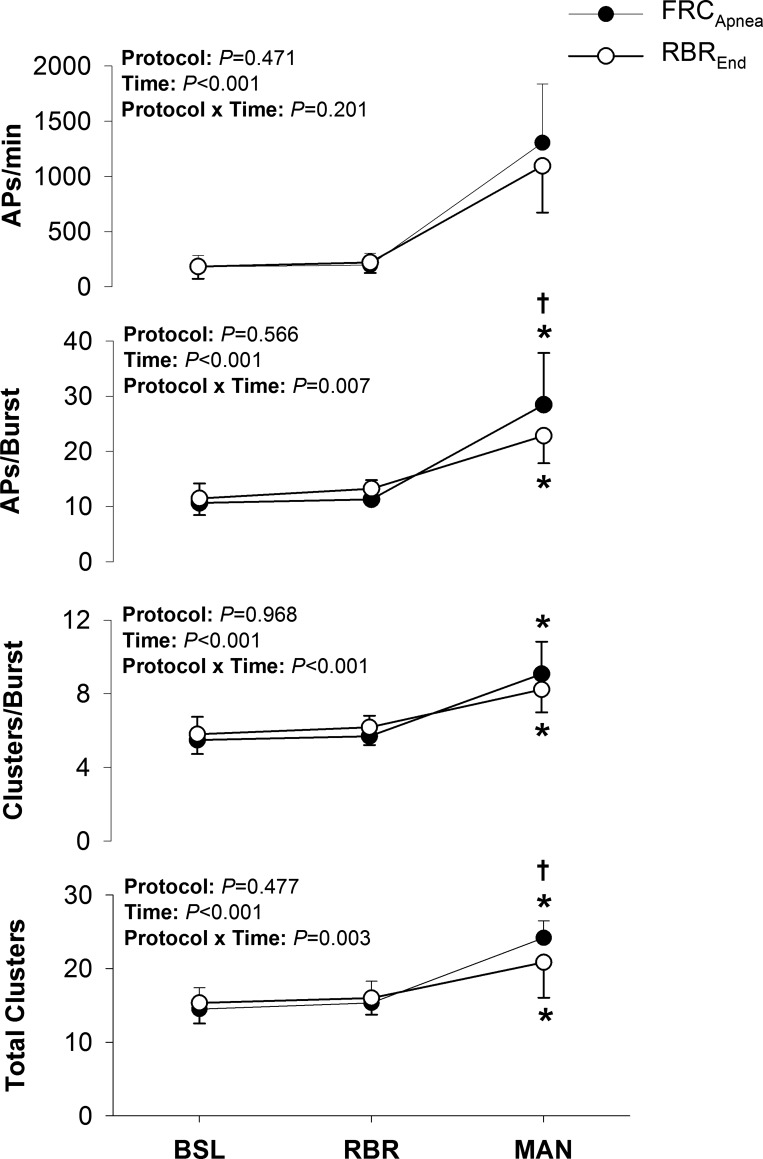
Sympathetic action potential (AP) recruitment during baseline (BSL), initial rebreathe (RBR), and maneuver (MAN) periods of the FRC_Apnea_ and RBR_End_ protocols. *P < 0.05, significantly different from BSL. †*P* < 0.01, significantly different from RBR_End_.

Sympathetic AP discharge indexes during the baseline, rebreathe, and maneuver periods of the FRC_Apnea_ and RBR_End_ protocols are displayed in Fig. 3. Compared with baseline, AP frequency (FRC_Apnea_: +1122 ± 534 APs/min; RBR_End_: +912 ± 429 APs/min; *P* < 0.001) increased during both maneuvers; however, AP frequency was not different during FRC_Apnea_ and RBR_End_ (1,304 ± 534 vs. 1,095 ± 423 APs/min; *P* = NS). Once again, the increased AP frequency was due to the elevated burst frequency but also to an increase in the AP content per integrated sympathetic burst (FRC_Apnea_: +18 ± 7 APs/burst; RBR_End_: +11 ± 5 APs/burst; both *P* < 0.001) during both maneuvers, compared with baseline. However, the AP content per integrated sympathetic burst was greater during FRC_Apnea_ than during RBR_End_ (28 ± 9 vs. 23 ± 5 APs/burst; *P* < 0.05). Furthermore, when APs were binned according to peak-to-peak amplitude (i.e., into clusters), the number of total AP clusters increased during FRC_Apnea_ (+10 ± 2 total clusters; *P* < 0.001) and RBR_End_ (+6 ± 4 total clusters; *P* < 0.001), yet total AP clusters was greater during FRC_Apnea_ (*P* < 0.01). Finally, the number of active clusters per burst (FRC_Apnea_: +4 ± 2 clusters/burst; RBR_End_: +2 ± 1 clusters/burst; both *P* < 0.001) was elevated above baseline levels during both maneuvers, whereas no differences were observed during FRC_Apnea_ vs. RBR_End_ (*P* = NS).

The hemodynamic responses to FRC_Apnea_ and RBR_End_ are shown in Table 3. Compared with baseline, MAP (FRC_Apnea_: +18 ± 9 mmHg; RBR_Matched_: +20 ± 12 mmHg; both *P* < 0.001) increased similarly during both maneuvers (*P* = NS). However, this pressor response was achieved differently for FRC_Apnea_ and RBR_End_. Specifically, compared with baseline, TPR increased during FRC_Apnea_ (*P* < 0.001) but not during RBR_End_ (*P* = NS), whereas CO increased during RBR_End_ (*P* < 0.01) but decreased during FRC_Apnea_ (*P* < 0.001). The latter was due to an increase in HR during RBR_End_ (*P* < 0.001) compared with no change during FRC_Apnea_ (*P* = NS) and a similar decrease in SV during both maneuvers (both *P* < 0.001).

#### FRC-RBR_ALT_.

**Fig. 4.**
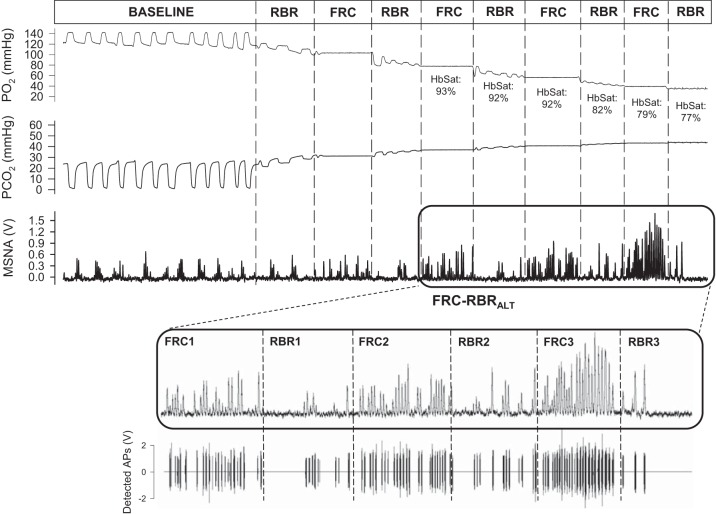
Representative recordings of the integrated muscle sympathetic nerve activity (MSNA) neurogram and detected action potentials (APs) (and associated chemoreflex stimuli) from one individual during the alternating functional residual capacity (FRC) apnea and rebreathe (RBR) protocol (FRC-RBR_ALT_). Po_2_, partial pressure of oxygen; Pco_2_, partial pressure of carbon dioxide; HbSat, hemoglobin saturation. Boxed area represents the portion of the maneuver used for analysis (see *insets*).

Figure 4 displays representative recordings of the integrated MSNA neurogram and detected APs (and associated chemoreflex stimuli) from one individual during the FRC-RBR_ALT_ protocol. HbSat levels were decreased compared with baseline during each of the final two FRC and RBR periods of the FRC-RBR_alt_ protocol (Table 5; all *P* < 0.001). Compared with baseline, $PET_{CO_{2}}$ was increased and $PET_{O_{2}}$decreased during each FRC and RBR period (Table 5; all *P* < 0.001). No differences existed between corresponding periods of FRC and RBR for HbSat or $PET_{CO_{2}}$and $PET_{O_{2}}$levels (all *P* = NS).

**Table 5.** Hemodynamic responses to the FRC-RBR_alt_ protocol

	Baseline	FRC_1_	RBR_1_	FRC_2_	RBR_2_	FRC_3_	RBR_3_
MAP, mmHg	88 ± 3	92 ± 3	88 ± 3	96 ± 7	96 ± 6	105 ± 11*	101 ± 10*
HR, beats/min	63 ± 6	57 ± 7	63 ± 7	60 ± 9	66 ± 11	58 ± 10†	69 ± 12
SV, ml	97 ± 23	88 ± 21	88 ± 21	87 ± 24	87 ± 24	90 ± 28	91 ± 26
CO, l/min	6.0 ± 1.1	5.0 ± 0.9*	5.4 ± 0.9*	5.1 ± 1.1*	5.6 ± 0.8	5.1 ± 1.0*†	6.1 ± 0.8
TPR, mmHg·l^−1^·min^−1^	15.1 ± 2.6	19.2 ± 3.9*	16.4 ± 2.2	20.0 ± 4.0*	17.6 ± 3.0	21.5 ± 5.6*†	17.0 ± 3.2
HbSat, %	99.5 ± 0.5	95.3 ± 2.3	95.3 ± 3.5	88.6 ± 5.5*	88.7 ± 6.9*	75.9 ± 2.9*	72.1 ± 11.9*
$PET_{CO_{2}}$, Torr	28 ± 7	40 ± 3*	41 ± 4*	44 ± 4*	44 ± 5*	47 ± 5*	48 ± 7*
$PET_{O_{2}}$, Torr	114 ± 12	67 ± 11*	69 ± 11*	50 ± 4*	50 ± 10*	40 ± 4*	36 ± 8*

Values are means ± SD. FRC, functional residual capacity apnea; RBR, rebreathe; MAP, mean arterial pressure; HR, heart rate; SV, stroke volume; CO, cardiac output; TPR, total peripheral resistance; HbSat, hemoglobin saturation; $PET_{CO_{2}}$, end-tidal partial pressure of carbon dioxide; $PET_{O_{2}}$, end-tidal partial pressure of oxygen.

* *P* < 0.001, significantly different from baseline.

† *P* < 0.001, significantly different from corresponding RBR period.

Table 6 displays the integrated MSNA indexes during baseline and FRC and RBR periods of the FRC-RBR_alt_ protocol. Specifically, compared with baseline, all integrated MSNA variables were elevated during each FRC (all *P* < 0.001), but not during any of the three RBR periods (all *P* = NS). As such, every corresponding FRC-RBR period was associated with greater levels of integrated MSNA during FRC compared with RBR (all *P* < 0.001).

**Table 6.** Integrated MSNA responses to the FRC-RBR_alt_ protocol

	Baseline	FRC_1_	RBR_1_	FRC_2_	RBR_2_	FRC_3_	RBR_3_
Burst frequency, bursts/min	18 ± 7	40 ± 7*†	14 ± 7	41 ± 6*†	20 ± 7	53 ± 8*†	19 ± 8
Burst incidence, bursts/100 hb	29 ± 10	69 ± 9*†	22 ± 12	69 ± 13*†	30 ± 10	91 ± 8*†	27 ± 9
Burst amplitude, AU	54 ± 12	75 ± 18*	54 ± 7	92 ± 13*†	62 ± 9	135 ± 28*†	80 ± 18*
Total MSNA, AU/min	931 ± 327	2,997 ± 969*†	726 ± 339	3,760 ± 827*†	1,262 ± 515	7,076 ± 1,498*†	1,427 ± 540

Values are means ± SD. MSNA, muscle sympathetic nerve activity; FRC, functional residual capacity apnea; RBR, rebreathe; hb, heart beats; AU, arbitrary units.

* *P* < 0.001, significantly different from baseline.

† *P* < 0.001, significantly different from corresponding RBR period.

**Fig. 5.**
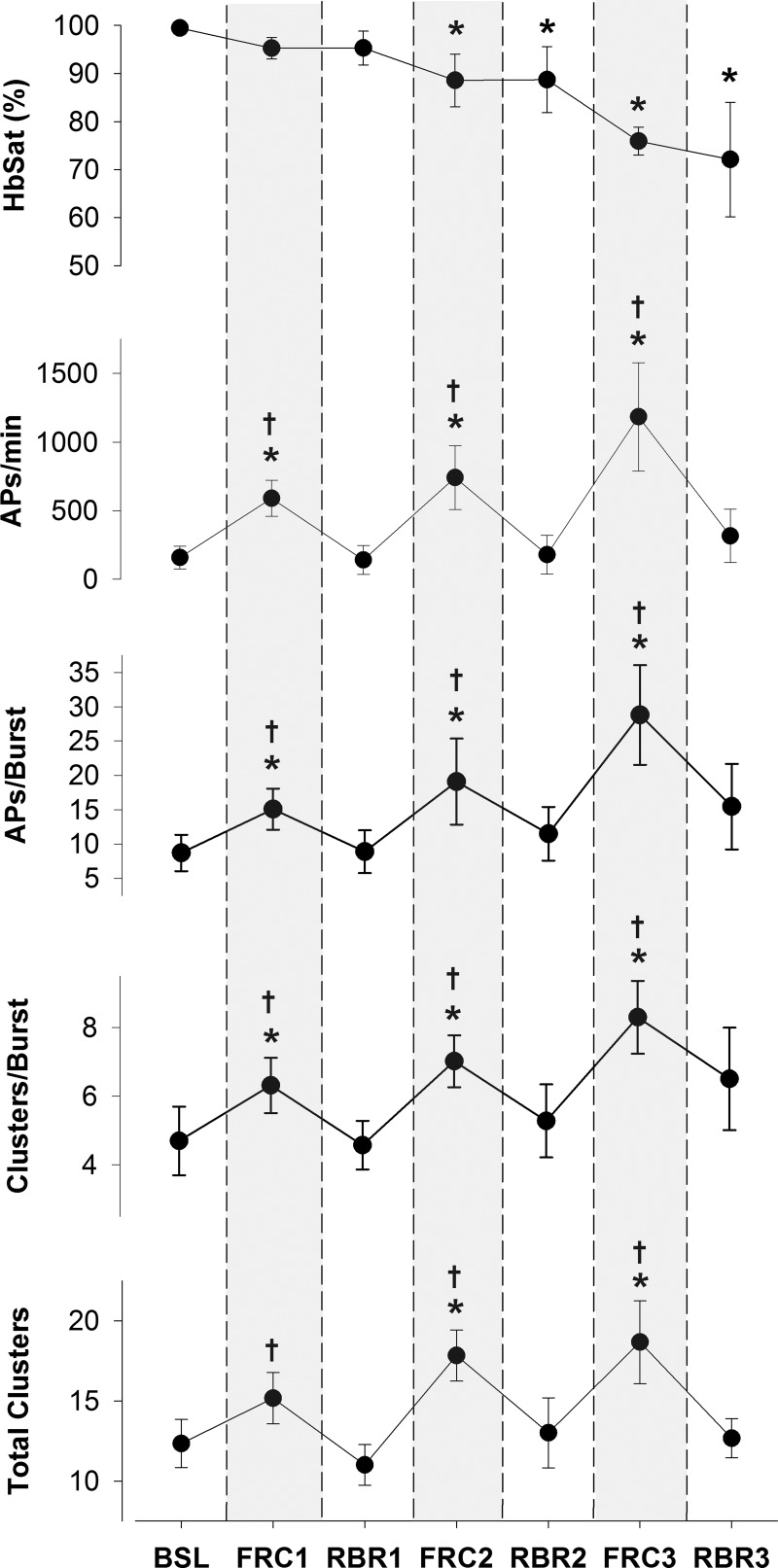
Sympathetic action potential (AP) recruitment during baseline (BSL) and alternating periods of apnea at functional residual capacity (FRC) and rebreathe (RBR). HbSat, hemoglobin saturation. *P < 0.001, significantly different from BSL. †*P* < 0.001, significantly different from corresponding RBR.

Sympathetic AP discharge indexes at baseline and during the FRC and RBR periods of the FRC-RBR _alt_ protocol are shown in Fig. 5. Compared with baseline, all AP indexes were increased during each FRC (all *P* < 0.001), but not during any RBR period (all *P* = NS). Therefore, every corresponding FRC-RBR period was associated with greater AP recruitment during FRC than during RBR (all *P* < 0.001).

Finally, the hemodynamic responses to the FRC-RBR_alt_ protocol are provided in Table 5. Compared with baseline, MAP increased during the final FRC and RBR periods (both *P* < 0.001), whereas TPR increased during each FRC (all *P* < 0.001), but not during RBR (all *P* = NS). HR was reduced during the final FRC compared with the final RBR (*P* < 0.001), whereas SV remained unchanged throughout the FRC-RBR_alt_ protocol (all *P* = NS). CO decreased during each FRC (*P* < 0.001) and was above baseline levels during the first RBR period (*P* < 0.001), but not in each of the final two RBR periods (both *P* = NS).

## DISCUSSION

The current study was designed to study the influences of chemoreceptor stimuli (i.e., hypoxia and hypercapnia) and ventilation on sympathetic AP recruitment patterns during chemoreflex stress. First, we demonstrated that during similar levels of (high) chemoreflex stress, the act of ventilation per se restrained the increased firing of already active, lower threshold axons and, furthermore, inhibited the recruitment of latent, higher threshold axons that appeared during apnea. Second, recruitment of previously silent axons was observed during severe chemoreflex stimulation induced by the continued rebreathe protocol; however, sympathetic AP recruitment was still restrained compared with apnea. Finally, during alternating periods of apnea and rebreathe, the act of rebreathing between apneas was associated with a rapid and complete withdrawal of sympathetic AP recruitment, despite the ever-increasing chemoreflex stimulus. As such, it appears that the sympathoinhibitory effect of ventilation during chemoreceptor activation affects both the frequency of neuronal discharge and recruitment of latent axons, even at severe levels of chemoreflex stress.

The sympathoexcitatory response to voluntary apnea is robust and includes a powerful stimulus for efferent sympathetic axonal recruitment (Badrov et al. 2015, 2016a; Breskovic et al. 2011; Seitz et al. 2012; Steinback et al. 2010b). The mechanisms mediating this sympathetic response are expected to include chemoreflex activation whereby both hypoxia and hypercapnia produce large increases in total sympathetic nerve activity (Saito et al. 1988; Somers et al. 1989a). Nevertheless, the absence of ventilation during apnea augments the sympathetic drive to chemoreflex activation (Somers et al. 1989a; Steinback et al. 2010a). Under baseline conditions, ventilation exerts a powerful within-breath modulator of sympathetic neural recruitment (Seals et al. 1990) whereby deep breathing exposes the increased and decreased MSNA during expiration and inspiration, respectively (Eckberg et al. 1985). Nonetheless, over multiple breaths, MSNA inhibition during inspiration was compensated for by augmented burst amplitude during expiration (Seals et al. 1990). Therefore, the pattern of MSNA was affected by ventilation, but not the total MSNA response. Previously, Seitz et al. (2013) illustrated the dominating ability of ventilation to inhibit the large sympathetic response to a ~30-s apnea and further showed that this inhibitory effect was not due to chemoreflex cessation. Therefore, mechanisms mediating the large increase in sympathetic outflow during an apnea are complex and apparently not dependent entirely on chemoreflex stimulation. In the current analysis, the sympathetic response to apnea might also be aided by a modest reduction in cardiac output due to bradycardia (see Table 1). Of note, obtained through pulse pressure contour analysis, CO represents an indirect measurement of CO in this study.

In addition to the study of total MSNA levels, the current study explored the ability of ventilation to modify AP recruitment patterns through a large range of chemoreflex stimulation in elite divers. The current results support the conjecture that ventilation modulates sympathetic postganglionic axonal recruitment and extend this conclusion to include high/severe levels of chemoreflex stress. Specifically, replicating earlier studies (Badrov et al. 2015, 2016a; Steinback et al. 2010b), the large increases in neural outflow during apnea were associated with increases in the within-burst firing frequency of already recruited sympathetic fibers, as well as the recruitment of latent (i.e., not present at baseline) subpopulations of larger sized axons. However, for similar levels of hypoxia and hypercapnia, these neural recruitment patterns were not achieved in the presence of ventilation (i.e., during RBR_Matched_). Indeed, RBR_Matched_ was associated with a lower firing frequency and a restrained increase in the number of APs per integrated burst such that, overall, sympathetic AP frequency increased ~700% during FRC_Apnea_ but only ~300% during RBR_Matched_. Furthermore, the capacity of the sympathetic nervous system to recruit latent neural subpopulations was inhibited during RBR_Matched_, despite the same chemoreflex load, indicating the profound effect of ventilation on AP recruitment patterns. Therefore, these data replicate earlier observations (Seitz et al. 2013) but at the extremes for human volitional chemoreflex stress tolerance.

Nonetheless, the current data do support the idea that the sympathoinhibitory effect of ventilation is less effective at more severe levels of chemoreflex stress. Specifically, latent axons were recruited during the more severe chemoreflex stress induced by the continued rebreathe protocol, despite the presence of ventilation (i.e., RBR_End_). Interestingly, in this scenario (i.e., FRC_Apnea_ vs. RBR_End_) the total integrated MSNA response was similar between protocols, but the underlying patterns of AP recruitment differed by maneuver. These data highlight a challenge associated with quantification of MSNA based on burst metrics.

The protocol alternating between FRC and rebreathing further enforces the role of ventilation in AP recruitment, across many levels of progressive chemoreflex stress. In this case, all occurrences of rebreathe, despite no relief of chemical drive, were associated with an inhibition of sympathetic AP discharge to baseline levels. Specifically, whereas each apnea was associated with increased within-burst firing of lower threshold axons and recruitment of latent, larger sized axons, each subsequent period of rebreathe was accompanied by a total withdrawal of this sympathetic AP recruitment. This withdrawal of sympathetic drive following the resumption of breathing in the face of similar and/or elevated chemical drive is consistent with findings from the integrated MSNA neurogram (Seitz et al. 2013; Steinback et al. 2010a; Watenpaugh et al. 1999). Of note, Seitz et al. (2013) demonstrated an immediate inhibition of MSNA following the break point of end-expiratory apnea, which was not influenced by whether subjects inhaled room air or asphyxic gas on the resumption of breathing.

Therefore, the present data support previous findings that indicate ventilation per se is responsible for the sympathoinhibition across a wide range of chemoreflex stress. However, the potency of this inhibitory effect appears to depend on the temporal or situational conditions. For example, acute periods of rebreathe following apnea were associated with complete withdrawal of AP discharge. However, when ventilation continued against a background of progressive chemoreflex stress (i.e., RBR_End_ protocol), sympathetic axonal recruitment did occur over time, albeit at levels that were less than those observed during apnea. These observations suggest that the sympathoinhibitory influence of breathing may be robust acutely but, perhaps, less effective over time even as the chemoreflex load continues to extreme levels.

The mechanisms underlying the ventilatory restraint of sympathetic axonal recruitment during high levels of chemoreflex stress remain unknown and cannot be elucidated from the current results. Possible contributors include lung-stretch receptor feedback (Seitz et al. 2013; Somers et al. 1989b) and perceptual factors associated with the stress experienced during prolonged breath-holding (Fowler 1954; Heusser et al. 2009). Stated differently, the volitional effort not to breathe coupled with the growing desire to breathe during voluntary apnea may result in a greater perception of stress than that of rebreathing, thereby influencing sympathetic drive. Callister et al. (1992) have shown that levels of perceived stress influence directly the sympathetic response during cognitive challenge. However, stress-induced sympathetic responses related to such prolonged breath-holds and chemoreflex duress produced by these elite breath-hold competitors have not been quantified. Nonetheless, components of the current observations argue against a “stress” determinant of sympathetic drive during apnea. First, all participants were instructed to continue each protocol until they achieved ~85% of their self-perceived “maximal” tolerance for the maneuver. As such, the FRC_Apnea_ and RBR_End_ protocols were “matched” for levels of perceived stress whereby greater AP recruitment was still observed during apnea. Second, we deliberately studied breath-hold divers who are uniquely trained to endure this level of stress and perform these maneuvers often. Thus, the fact that these were not “maximal” efforts, combined with the participants’ familiarity with the maneuvers, likely suggests that levels of perceived stress during voluntary apnea were not maximal. However, we acknowledge that the perceptual levels of stress magnitude in sympathetic activation in the context of apneas are not known. Finally, albeit in a volitional handgrip contraction and metaboreflex activation model, we have found that a central perceptual component has little to do with actual axonal recruitment, as opposed to the effects of peripheral-reflex mechanisms (Badrov et al. 2016b). Therefore, we do not regard a specific role of perceptual factors in the current results as important in the observations. We also do not consider baroreflex mechanisms to have contributed to the divergent sympathetic recruitment responses between conditions. Specifically, the differing AP recruitment responses persisted despite large and similar (or slightly greater during apnea) increases in MAP during both maneuvers. Therefore, it is likely that resetting of the arterial baroreflex occurred during both maneuvers (Muenter Swift et al. 2003), the magnitude of which might be greater during apnea. Nonetheless, ventilation appears to modulate sympathetic axonal recruitment during high, and even severe, chemoreflex stress. The mechanisms mediating this restraint on sympathetic neural recruitment will require additional study.

In summary, the present study demonstrated that the sympathetic axonal recruitment strategies that are normally observed during apnea are largely restrained by the act of ventilation despite similar and/or greater levels of high, and even extreme, chemoreflex stress. Specifically, during severe levels of hypoxia and hypercapnia that exert powerful sympathoexcitatory stimuli (Hardy et al. 1994; Saito et al. 1988; Somers et al. 1989a), both the frequency of within-burst firing of already active sympathetic fibers and the recruitment of subpopulations of previously silent, higher threshold axons are restrained in the presence of ventilation.

## GRANTS

This study was supported by Croatian Science Foundation Grant IP-2014-1937 (O. F. Barak, I. Drvis, Z. Dujic) and Natural Sciences and Engineering Research Council of Canada Grant 217916-2013 (J. K. Shoemaker). M. B. Badrov was supported by a Canadian Institutes of Health Research Doctoral Scholarship Award and a Canada Graduate Scholarships – Michael Smith Foreign Study Supplement Award. J. K. Shoemaker is a Tier 1 Canada Research Chair.

## DISCLOSURES

No conflicts of interest, financial or otherwise, are declared by the authors.

## AUTHOR CONTRIBUTIONS

M.B.B., O.F.B., I.D., Z.D., and J.K.S. conceived and designed research; M.B.B., O.F.B., T.M., M.L., I.D., Z.D., and J.K.S. performed experiments; M.B.B., L.N.S., and L.J.B. analyzed data; M.B.B., Z.D., and J.K.S. interpreted results of experiments; M.B.B. and L.J.B. prepared figures; M.B.B. drafted manuscript; M.B.B., O.F.B., T.M., L.N.S., L.J.B., Z.D., and J.K.S. edited and revised manuscript; M.B.B., O.F.B., T.M., L.N.S., L.J.B., M.L., I.D., Z.D., and J.K.S. approved final version of manuscript.
